# Anxiety and distress following receipt of results from routine HPV primary testing in cervical screening: The psychological impact of primary screening (PIPS) study

**DOI:** 10.1002/ijc.32540

**Published:** 2019-07-23

**Authors:** Emily McBride, Laura A.V. Marlow, Alice S. Forster, Deborah Ridout, Henry Kitchener, Julietta Patnick, Jo Waller

**Affiliations:** ^1^ Research Department of Behavioural Science and Health, Institute of Epidemiology and Health Care University College London London United Kingdom; ^2^ Population, Policy and Practice Programme UCL Great Ormond Street Institute of Child Health London United Kingdom; ^3^ Women's Cancer Centre, Institute of Cancer Sciences University of Manchester Manchester United Kingdom; ^4^ Cancer Epidemiology Unit, Nuffield Department of Population Health University of Oxford Oxford United Kingdom

**Keywords:** psychological impact, cancer screening, human papillomavirus, women, psychological wellbeing

## Abstract

We used a cross‐sectional survey to examine short‐term anxiety and distress in women receiving different results following routine human papillomavirus (HPV) primary testing at cervical screening. Participants were women aged 24–65 (*n* = 1,127) who had attended screening at one of five sites piloting HPV primary screening in England, including a control group with normal cytology who were not tested for HPV. Women completed a postal questionnaire ~2 weeks after receiving their screening result. Unadjusted mean anxiety scores ranged from 32.9 (standard deviation [SD] = 12.2) in HPV‐negative women to 42.1 (SD = 14.9) in women who were HPV‐positive with abnormal cytology. In adjusted analyses, anxiety was significantly higher in women testing HPV‐positive with either normal cytology (mean difference [MD] = 3.5, CI: 0.6–6.4) or abnormal cytology (MD = 7.2, CI: 3.7–10.6), than the control group. Distress was slightly higher in women who tested HPV‐positive with abnormal cytology (MD = 0.9, CI: 0.02–1.8), than the control group. We also found increased odds of very high anxiety in women who tested HPV‐positive with normal or abnormal cytology compared to the control group. This pattern of results was only observed among women receiving their first HPV‐positive result, not among women found to have persistent HPV at 12‐month follow‐up. Testing HPV‐positive with normal cytology for the first time, is associated with elevated anxiety despite carrying very low immediate cervical cancer risk. However, receiving the same test result at 12‐month early recall does not appear to be associated with higher anxiety, suggesting anxiety may normalise with repeated exposure and/or over time.

## Abstract 2

**What's new?**

While a positive test for human papillomavirus (HPV) with negative cytology is associated with low cervical cancer risk, recall for additional HPV testing may have undesirable psychological impacts. In this study, involving women who participated in HPV primary screening in England, women who were informed of an HPV‐positive result for the first time experience increased anxiety and concern. Meanwhile, positive HPV testing at 12‐month recall had little adverse psychological impact, suggesting that anxiety concerning a positive HPV result is relatively short‐lived. Nonetheless, strategies should be in place to mitigate anxiety in women receiving a positive result for the first time.

**Abbreviations**
ANOVA: analysis of variance
GHQ: General Health Questionnaire
HPV: human papillomavirus
HRA: health research authority
IMD: index of multiple deprivation
MD: mean difference
NHS: National Health Service
NHSCSP: National Health Service Cervical Screening Programme
OR: odds ratio
SD: standard deviation
S‐STAI: short‐form state‐trait anxiety inventory

## Introduction

Over 3 million women take part in the National Health Service Cervical Screening Programme (NHSCSP) in England every year.^1^ In the UK and elsewhere, cervical screening is changing to incorporate primary human papillomavirus (HPV) testing. Testing for the presence of high‐risk HPV has been shown to increase sensitivity for the detection of precancerous lesions and is predicted to prevent almost 500 additional cancers per year in England.^2^, ^3^, ^4^, ^5^, ^6^ Ahead of the roll‐out of HPV primary screening across England, the new programme has been piloted in six sentinel NHS sites.^7^ This provided the opportunity to evaluate the psychological impact of HPV primary screening prior to full implementation.

Psychological considerations are central to the successful implementation of HPV primary screening. Under the primary screening protocol, all women who attend will be told whether they test positive or negative for high‐risk HPV. Testing positive for HPV can lead to elevated anxiety, fear and concern related to the possible development of cervical cancer.^8^, ^9^, ^10^, ^11^, ^12^ When HPV testing is used to triage women with borderline or mildly abnormal cytology, anxiety and distress have been found to be higher in women testing positive for HPV than those who test negative or do not have an HPV test,^10^ although the difference seems relatively short‐lived.^13^ HPV can also carry a negative label due to its sexually transmitted nature, sometimes resulting in shame, stigma and concerns about fidelity and relationships.^11^, ^14^, ^15^ As well as testing far greater numbers of women for HPV, the primary screening protocol also creates a new group of women who have normal cytology, but test positive for high‐risk HPV. These women are at very low immediate risk of cervical cancer but are recalled at 12 months for repeat HPV testing. The prevalence of this result was 8.5% in the English HPV primary screening pilot^7^ which would mean around 270,000 women receiving it in England every year. Women's psychological response to this result in routine cervical screening is unknown.

We aimed to compare anxiety and distress between women receiving the different possible test results in HPV primary screening. The partial conversion of the pilot screening laboratories to HPV screening allowed us to compare these women with those receiving a normal result as part of the current cytology‐based programme.^16^ To the best of our knowledge, this is the first major study to quantitatively measure the short‐term psychological impact of HPV primary testing within a routine programme.

## Materials and Methods

### Design

A cross‐sectional between‐groups design was employed to assess women's psychological responses shortly after receiving their cervical screening test results (baseline), as well as 6 months, and 12 months later. This article reports baseline findings.

### Participants

Participants were women aged 24–65 years who had been screened in one of five NHS sites in England using HPV primary testing as part of the NHSCSP pilot: North West London, Sheffield, Norfolk and Norwich, Liverpool and Central Manchester. The NHS pilot sites had catchment areas with broad geographical coverage across England. Baseline recruitment to our study commenced on 18/11/2016 and ceased on 14/10/2017 with approximately 3–5 months of active recruitment per site. Health Research Authority (HRA) approval was granted on 26/09/2016 (Research Ethics Committee reference: 16/LO/0902 and Confidentiality Advisory Group reference: 16/CAG/0047).

Women were eligible if they had received one of six possible combinations of HPV and/or cytology test results within the last 2 weeks, as indicated by their NHS clinical records. The sampling strategy aimed to recruit roughly equal numbers of women from each test result group. Five of the groups were recruited from women included in the HPV primary screening pilot. We also included a control group who had received a normal cytology result at standard cytology‐based screening within the same geographical areas and processed by the same laboratories. Table 1 provides an overview of the six result groups. A full description of the recruitment procedure can be found in our protocol.^16^

**Table 1:** HPV and cytology results for the six groups included in the study

	HPV result	Cytology result
Group 1 (control)	Not tested	Normal
Group 2	Negative	Not tested
Group 3	Positive	Normal
Group 4	Positive	Abnormal
Group 5^1^	Persistent positive at 12 months	Normal
Group 6^1^	Negative at 12 months	Not tested

Table 1 has been adapted from our protocol paper.^16^

1 Women in Groups 5 and 6 had all tested HPV positive with normal cytology at their first HPV primary screen and were recruited to the study after their 12‐month follow‐up test.

### Procedures and clinical management

Prior to participant identification, women were notified about the study *via* a web link which was printed in HPV primary screening information leaflets and sent alongside their screening invitation letters. The link directed women to our university departmental website which provided study information as well as details of how to opt‐out of being approached to take part. No women opted out.

Eligible women were identified by staff in NHS cytology and virology departments at each of the five participating sites. Staff allocated each potential participant a unique identity number, which they recorded and linked to patient name, address, age, screening history, NHS site and test result; these outcomes will collectively be referred to as “NHS data.”

Potential participants were mailed invitation packs to their home, which contained an invitation letter, participant information sheet, consent form, questionnaire booklet and prepaid return envelope. To maximise the response rate, a reminder pack containing the same documents was mailed 3 weeks later.

Women opted to take part by returning their completed consent form and questionnaire to the university. All documents were preprinted with unique identity numbers which allowed questionnaire data to be linked with NHS data. At the end of recruitment, UCL received NHS data on all of the women approached (*n* = 5,494) in nonidentifiable format (name and address removed, and replaced with Index of Multiple Deprivation score and quintile) to allow for demographic comparisons between responders and nonresponders.

### Outcome measures

Primary outcomes were state anxiety and general distress (measured using the state‐trait anxiety inventory [S‐STAI‐6]^17^ and General Health Questionnaire [GHQ‐12],^18^ respectively). Secondary outcomes reported include very high anxiety (score > 49/80 on S‐STAI‐6), case‐level distress (score > 3/12 on GHQ‐12), self‐reported response to test results (concern and reassurance) and worry about developing cervical cancer. See Table 2 for a more detailed overview of the primary and secondary outcome measures.

**Table 2:** A summary of the primary and secondary outcomes measures

	Description	Scoring and interpretation
Anxiety (S‐STAI‐6)	The short‐form state anxiety inventory (S‐STAI‐6) is a six‐item, validated questionnaire measuring anxiety.^17^ Cronbach's alpha was 0.86 across all groups (*n* = 991), indicating a high level of internal consistency.	Scores range from 20 to 80.
		Normal score expected in the general population at 34–36.
		Very high anxiety at >49.
General distress (GHQ‐12)	The General Health Questionnaire (GHQ‐12) is a 12‐item validated questionnaire used to measure general distress.^18^ Cronbach's alpha was 0.91 across all groups (*n* = 1,106), indicating a high level of internal consistency.	Scores range from 0 to 12.
		Case‐level distress at >3.
Concern about test result^1^	Concern relating to test result was measured by asking: *How concerned do you feel about your recent screening result?* This question was adapted from the previous NHSCSP psychological evaluation.^10^	Five‐point Likert scale indicating:
		1 = not at all concerned.
		2 = slightly concerned.
		3 = somewhat concerned.
		4 = moderately concerned.
		5 = very concerned.
		Scores of 1–3 were classified as lower concern; scores of 4–5 were classified as higher concern.
Reassurance from test result^1^	Reassurance relating to test result was measured by asking: *How reassured do you feel about your recent screening result?* This question was adapted from the previous NHSCSP psychological evaluation.^10^	Five‐point Likert scale indicating
		1 = not at all reassured.
		2 = slightly reassured.
		3 = somewhat reassured.
		4 = moderately reassured.
		5 = very reassured.
		Scores of 1–2 were classified as lower reassurance; scores of 3–5 were classified as higher reassurance.
Worry about cervical cancer^1^	Worry about developing cervical cancer was measured by asking: *How worried are you about getting cervical cancer in the next 10 years?* This question was adapted from the previous NHSCSP psychological evaluation.^10^	Five‐point Likert scale indicating:
		1 = not at all worried.
		2 = slightly worried.
		3 = somewhat worried.
		4 = moderately worried.
		5 = very worried.
		Scores of 1–3 were classified as lower worry; scores of 4–5 were classified as higher worry.

Primary outcomes were anxiety and general distress. Secondary outcomes included concern, reassurance and worry.

1 Cut‐off points for high/low concern, reassurance and worry were based on the most stable estimates and distribution of participant responses; sensitivity analyses were performed comparing the different possible cut‐off points which revealed consistent findings.

Demographic characteristics based on self‐report were the highest level of education, ethnicity, marital status and HPV vaccine status. NHS data included information on age, cervical screening history (number of previous screens), NHS site and Index of Multiple Deprivation score and quintile (IMD; a marker of area‐level deprivation, based on residential postcode).^19^ Further details on descriptive outcomes can be found in our protocol paper.^16^

### Sample size and response rate

The study was powered to detect a small‐to‐medium between‐group difference (*f* = 0.14) in anxiety (as measured by the S‐STAI‐6) at the 12‐month time‐point.^10^ Based on previous studies,^10^, ^13^ we expected anxiety scores across groups to be in the range of 36–40, with a standard deviation (SD) of 12. With an α of 0.05, we calculated that a minimum sample size of 673 in total with roughly 112 per group would give us 80% power to detect between‐group differences in anxiety. We therefore initially planned to approach 3,415 participants anticipating a baseline response rate of 35%, with 75% of baseline responders returning a 6‐month follow‐up questionnaire and 75% of 6‐month responders returning a 12‐month follow questionnaire. However, as the study progressed, our response rate was lower than expected at approximately 22%. In line with our protocol,^16^ we increased the number of women approached to adjust for this (to *n* = 5,494). We estimated that approaching approximately 5,500 women would yield a total sample size of 1,210 at baseline, 908 at 6‐months and 681 at 12‐months. Our baseline sample was 1,148 at baseline.

### Data analysis

Ten per cent of data were checked independently for errors by a member of the research team who was not involved in the initial data entry. Error rates were substantially below the prespecified cut‐off for no further action to be taken (<1% error for all outcomes). Demographic characteristics were compared between the six groups using one‐way ANOVA and Chi‐squared tests as appropriate.

We compared the demographic characteristics of responders and nonresponders (including age, test results, number of previous screens, NHS site and IMD quintile) which revealed small variations. See Supporting Information Table S1 for an overview of nonresponder demographic characteristics. To adjust for the fact that our approached sample may not have been fully representative of the screening population in the pilot sites, we generated and applied population weights based on age group (24–34; 35–44; 45–54; 55–65) and IMD quintile within each test result group. With permission from the Office for Data Release, we used data from 955,387 women who attended HPV primary screening (and primary cytology for the control group) within the NHSCSP in the five sites included in our study in 2017–2018 to calculate the weights.

For each of the primary outcomes (anxiety and distress), we compared the mean scores between the six groups using univariate regression analysis. Further to this, multiple regression analysis was performed to adjust for confounding factors: age, IMD score, ethnicity, marital status, education, number of previous cervical screens and NHS site. Results are presented as mean difference (MD) compared to the control group, along with 95% confidence intervals. We also present descriptive mean values and standard deviations for each of the six groups. For the secondary outcomes (very high anxiety, case‐level distress, worry about cancer, concern and reassurance about results), we fitted both univariate and multiple logistic regression models adjusting for the same confounding factors. Results are presented as odds ratios, indicating the odds of the outcome for each of the groups relative to the control group, with 95% confidence intervals. Due to skewed responses to the concern and reassurance items in the control group, we used the HPV positive with normal cytology group as the reference category for analyses of these outcomes.

Data completeness was >96% for the majority of outcomes and factors, with the exception of anxiety (89%) and IMD (93%). We used multiple imputation assuming data were missing at random to account for missing data. The imputation model included primary outcomes and all sociodemographic factors, which we assumed included all predictors of missingness. The final models were derived by fitting a regression model including all confounders, and estimates were combined using Rubin's rules.^20^

Demographic characteristics have been presented using nonweighted data. All primary and secondary results have been adjusted using the weights described above and using the imputed data. Supporting Information Table S2 presents the results of the primary and secondary analyses using unweighted data. Analyses were carried out using Stata v15^21^ and SPSS v25,^22^ and *p*‐value <0.05 was considered statistically significant.

## Results

**Figure 1:**
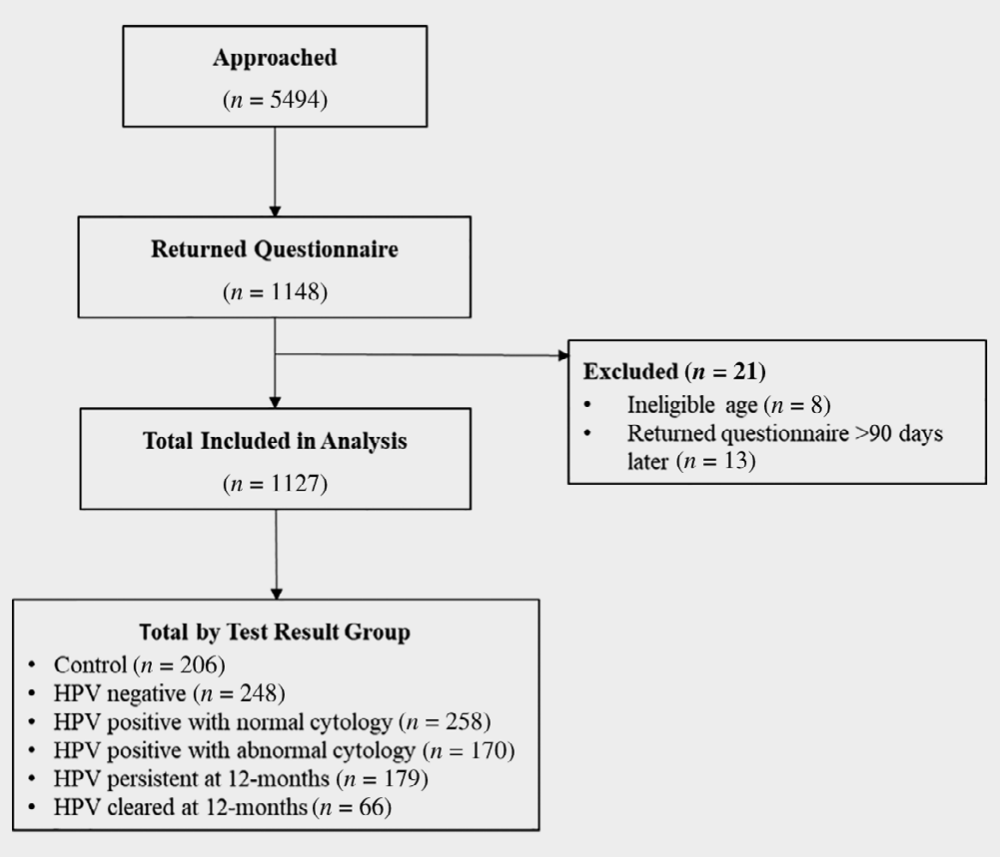
Overview of recruitment and response.

Five‐thousand‐four‐hundred‐ninety‐four women were invited to take part and 1,148 returned a questionnaire (response rate of 21%). Thirteen participants were excluded from the study due to returning a questionnaire over 90 days after date of identification and eight due to ineligible age (>65); 1,127 participants were included in the analyses. See Figure 1 for an overview of recruitment.

### Demographics

Table 3 shows unweighted demographic characteristics across the whole sample and by test result group. Overall, characteristics were similar across each of the test result groups, with some small differences relating to age, number of previous screens, marital status, IMD quintile and NHS site. These potential confounding variables were adjusted for in the analyses.

**Table 3:** Demographic characteristics of the whole sample (*n* = 1,127) and by results group (no weights or adjustments applied)

	Control (no HPV test)	HPV negative	HPV positive, normal	HPV positive, abnormal	HPV persistent at 12 months	HPV cleared at 12 months	Overall
*n* (%)	206 (18.3%)	248 (22.0%)	258 (22.9%)	170 (15.1%)	179 (15.9%)	66 (5.9%)	1,127 (100%)
Age (*n* = 1,125)							
Mean years (SD)	43.8 (11.0)	43.9 (11.4)	39.9 (12.2)	37.0 (10.6)	40.5 (12.0)	40.6 (11.7)	41.2 (11.8)
Marital status,^1^ *n* (%)							
Current partner	164 (80.8%)	214 (87.3%)	184 (72.4%)	111 (66.9%)	131 (74.9%)	51 (78.5%)	855 (77.2%)
No partner	39 (19.2%)	31 (12.7%)	70 (27.6%)	55 (33.1%)	44 (25.1%)	14 (21.5%)	253 (22.8%)
Ethnicity *n* (%)							
White (British or other)	180 (89.6%)	217 (88.6%)	235 (92.9%)	151 (91.0%)	167 (94.9%)	63 (96.9%)	1,013 (91.6%)
Other ethnicity	20 (10.0%)	27 (11.0%)	17 (6.7%)	15 (9.0%)	9 (5.1%)	2 (3.1%)	90 (8.1%)
Prefer not to say	1 (0.5%)	1 (0.4%)	1 (0.4%)	0 (0.0%)	0 (0.0%)	0 (0.0%)	3 (0.3%)
IMD Quintile, *n* (%)							
1 (most deprived)	42 (22.1%)	23 (10.0%)	46 (19.0%)	24 (15.6%)	25 (15.1%)	10 (16.7%)	170 (16.3%)
2	38 (20.0%)	46 (19.9%)	55 (22.7%)	33 (21.4%)	28 (16.9%)	11 (18.3%)	211 (20.2%)
3	44 (23.2%)	69 (29.9%)	53 (21.9%)	40 (26.0%)	53 (31.9%)	17 (28.3%)	276 (26.5%)
4	27 (14.2%)	40 (17.3%)	54 (22.3%)	30 (19.5%)	31 (18.7%)	11 (18.3%)	193 (18.5%)
5 (least deprived)	39 (20.5%)	53 (22.9%)	34 (14.0%)	27 (17.5%)	29 (17.5%)	11 (18.3%)	193 (18.5%)
Education, *n* (%)							
Degree or higher	91 (45.0%)	100 (41.2%)	109 (43.6%)	72 (43.9%)	76 (43.7%)	30 (46.2%)	478 (43.5%)
Qualification below degree	92 (45.5%)	126 (51.9%)	124 (49.6%)	82 (50.0%)	83 (47.7%)	30 (46.2%)	537 (48.9%)
No formal qualifications^2^	19 (9.4%)	17 (7.0%)	17 (6.8%)	10 (6.1%)	15 (8.6%)	5 (7.7%)	83 (7.6%)
No. of previous screens (*n* = 1,077)							
Mean screens (SD)	6.8 (4.7)	6.6 (4.4)	5.9 (5.1)	4.8 (4.7)	7.2 (5.5)	6.9 (5.0)	6.3 (4.9)
HPV vaccine status, *n* (%)							
1–3 doses	10 (5.0%)	10 (4.1%)	22 (8.9%)	18 (10.8%)	6 (3.5%)	1 (1.5%)	67 (6.1%)
NHS site, *n* (%)							
Liverpool	18 (8.7%)	47 (19.0%)	51 (19.8%)	24 (14.1%)	41 (22.9%)	2 (3.0%)	183 (16.2%)
Sheffield	23 (11.2%)	46 (18.5%)	47 (18.2%)	29 (17.1%)	54 (30.2%)	13 (19.7%)	212 (18.8%)
London North West	23 (11.2%)	39 (15.7%)	27 (10.5%)	31 (18.2%)	18 (10.1%)	9 (13.6%)	147 (13.0%)
Norfolk and Norwich	26 (12.6%)	30 (12.1%)	37 (14.3%)	34 (20.0%)	37 (20.7%)	36 (54.5%)	200 (17.7%)
Manchester	116 (56.3%)	86 (34.7%)	96 (37.2%)	52 (30.6%)	29 (16.2%)	6 (9.1%)	385 (34.2%)

Total *n* can be found in the end column for the categorical variables.

1 Marital status: current partner (married, civil partnership, living with partner, in a relationship) and no partner (single, divorced, widowed).

2 No formal qualifications included those with no qualifications and those who were still studying.

### Primary outcomes

#### *Anxiety (S‐STAI‐6)*

Regression analysis revealed statistically significant differences in anxiety between test result groups. Women who tested HPV positive with normal cytology or abnormal cytology had higher mean anxiety scores than women in the control group (no HPV test; MD = 3.5, 95% CI: 0.6–6.4, *p* = 0.02, and MD = 7.2, 95% CI: 3.7–10.6, *p* < 0.001, respectively). There were no differences in mean anxiety scores between the other groups and the control group.

#### *General distress (GHQ‐12)*

Regression analysis also revealed a higher mean general distress score for the HPV positive with abnormal cytology group compared to the control group (MD = 0.9, 95% CI 0.02–1.8, *p* < 0.04). There were no differences in general distress between the other groups and the control group. Tables 4 and 5 provide an overview of the results for anxiety and distress.

**Table 4:** Descriptive characteristics for primary and secondary outcomes by results group (no weights or adjustments applied)

	Control (no HPV test)	HPV negative	HPV positive, normal	HPV positive, abnormal	HPV persistent at 12 months	HPV cleared at 12 months	Overall
Anxiety							
Mean (SD)	34.9 (12.5)	32.9 (12.2)	38.3 (14.3)	42.1 (14.9)	36.8 (13.1)	37.0 (12.1)	36.7 (13.6)
*n* (%)	185 (18.4%)	232 (23.1%)	224 (22.3%)	148 (14.7%)	157 (15.6%)	60 (6.0%)	1,006 (100%)
Distress							
Mean (SD)	2.3 (3.3)	1.9 (3.0)	2.7 (3.6)	3.3 (3.8)	2.5 (3.2)	2.5 (3.7)	2.5 (3.4)
*n* (%)	204 (18.3%)	244 (21.9%)	257 (23.1%)	167 (15.0%)	177 (15.9%)	65 (5.8%)	1,114 (100%)
Very high anxiety							
Score > 49	25 (13.5%)	31 (13.4%)	50 (22.3%)	52 (35.1%)	28 (17.8%)	11 (18.3%)	197 (19.6%)
Score ≤ 49	160 (86.5%)	201 (86.6%)	174 (77.7%)	96 (64.9%)	129 (82.2%)	49 (81.7%)	809 (80.4%)
Case‐level distress							
Score > 3	49 (24.0%)	53 (21.6%)	71 (27.5%)	53 (31.5%)	50 (28.2%)	16 (24.2%)	292 (26.1%)
Score ≤ 3	155 (76.0%)	192 (78.4%)	187 (72.4%)	115 (69.0%)	127 (71.8%)	50 (75.8%)	826 (73.9%)
Worry about cancer							
Higher worry	30 (14.7%)	33 (13.4%)	114 (44.4%)	78 (46.2%)	78 (44.1%)	11 (16.7%)	344 (30.7%)
Lower worry	174 (85.3%)	213 (86.6%)	143 (55.6%)	91 (53.8%)	99 (55.9%)	55 (83.3%)	775 (69.3%)
Concern							
Higher concern	7 (3.4%)	7 (2.9%)	84 (32.7%)	79 (46.5%)	56 (31.5%)	3 (4.5%)	236 (21.1%)
Lower concern	198 (96.6%)	238 (97.1%)	173 (67.3%)	91 (53.5%)	122 (68.5%)	63 (95.5%)	885 (78.9%)
Reassurance							
Higher reassurance	186 (90.7%)	220 (89.8%)	108 (42.0%)	76 (45.0%)	80 (45.2%)	54 (81.8%)	724 (64.7%)
Lower reassurance	19 (9.3%)	25 (10.2%)	149 (58.0%)	93 (55.0%)	97 (54.8%)	12 (18.2%)	395 (35.3%)

All binary variables are presented as numbers (%) by test result group.

Abbreviation: SD, standard deviation.

**Table 5:** Results for primary and secondary outcomes by test result groups (weighted and adjusted)

	Control (no HPV test)	HPV negative	HPV positive, normal cytology	HPV positive, abnormal cytology	HPV persistent at 12 months	HPV cleared at 12 months
Anxiety						
MD (95% CI)	Ref	−1.1 (−3.9, 1.8)	**3.5 (0.6, 6.4)**	**7.2 (3.7, 10.6)**	2.1 (−1.1, 5.3)	1.0 (−3.3, 5.3)
*p*‐value		0.45	**0.02**	**<0.001**	0.21	0.65
Distress						
MD (95% CI)	Ref	−0.2 (−0.9, 0.4)	0.6 (−0.1, 1.3)	**0.9 (0.02, 1.8)**	0.1 (−0.7, 0.9)	0.2 (−1.1, 1.6)
*p*‐value		0.49	0.11	**0.04**	0.81	0.74
Very high anxiety						
Odds ratio (95% CI)	Ref	1.3 (0.7, 2.4)	**1.9 (1.1, 3.5)**	**3.5 (1.9, 6.6)**	1.4 (0.7, 2.8)	1.2 (0.5, 2.9)
*p*‐value		0.43	**0.03**	**<0.001**	0.31	0.66
Case‐level distress						
Odds ratio (95% CI)	Ref	1.0 (0.6, 1.7)	1.4 (0.9, 2.3)	1.4 (0.8, 2.4)	1.2 (0.7, 2.1)	1.0 (0.4, 2.3)
*p*‐value		0.92	0.17	0.25	0.48	0.99
Worry about cancer						
Odds ratio (95% CI)	Ref	1.1 (0.6, 2.1)	**4.8 (2.8, 7.9)**	**4.9 (2.7, 8.8)**	**5.0 (2.8, 8.9)**	0.90 (0.4, 2.1)
*p*‐value		0.67	**<0.001**	**<0.001**	**<0.001**	0.83
High concern						
Odds ratio (95% CI)	**0.05 (0.02, 0.1)**	**0.07 (0.03, 0.2)**	Ref^1^	**1.8 (1.2, 2.9)**	1.1 (0.7, 1.8)	**0.10 (0.02, 0.5)**
*p*‐value	**<0.001**	**<0.001**		**0.01**	0.60	**<0.01**
High reassurance						
Odds ratio (95% CI)	**12.0 (6.6, 21.7)**	**10.9 (6.3, 18.7)**	Ref^1^	1.3 (0.8, 2.1)	1.3 (0.8, 2.0)	**5.7 (2.4, 13.1)**
*p*‐value	**<0.001**	**<0.001**		0.24	0.3	**<0.001**

*p* ≤ 0.05 interpreted as statistically significant (shown in bold). Adjusted for age, marital status, ethnicity, index of multiple deprivation (IMD), education, number of previous screens and NHS site. Weighted by age group and IMD quintile.

1 The reference group for concern and reassurance is HPV positive with normal cytology due to very low and very high proportions (respectively) of positive responses in the control group for these two outcomes.

Abbreviations: 95% CI, 95% confidence intervals; MD, mean difference; Ref, reference group.

### Secondary outcomes

#### *Very high anxiety and case‐level general distress*

Logistic regression was performed to compare the odds of having very high anxiety scores (S‐STAI‐6 score > 49/80) between the results groups. We found significantly increased odds of very high anxiety in the HPV positive with normal cytology group (OR 1.9, 95% CI: 1.1–3.5) and HPV positive with abnormal cytology group (OR: 3.5, 95% CI: 1.9–6.6), compared to the control group (no HPV test). None of the other groups differed significantly from the control group.

Logistic regression was also performed to determine the effects of test result group on case‐level general distress (GHQ‐12 score > 3/12). None of the groups differed significantly from the control group.

#### *Worry about developing cervical cancer*

We used logistic regression to ascertain the effects of receiving different test results on the likelihood that women scored highly for worry about developing cervical cancer in the next 10 years (worry score > 3; moderately/very worried). After adjusting for potential confounding factors, all three HPV positive groups were found to be at significantly increased odds of high worry when compared to the control group (no HPV test), all with odds ratios over 4 (see Table 5).

#### *Concern and reassurance related to results*

Logistic regression was also performed to ascertain the effects of receiving different test results on the likelihood that women scored highly for concern (score > 3; moderately/very concerned) and highly for reassurance (score > 2; somewhat/moderately/very reassured). After adjusting for potential confounding factors, the odds of high concern in the HPV positive with abnormal cytology group was 1.8 (95% CI: 1.2–2.9) when compared to the HPV positive with normal cytology group. The odds of high concern were significantly lower for the three normal results groups (control, HPV negative and HPV cleared) when compared to the HPV positive with normal cytology group. All three normal results groups had significantly higher odds of high reassurance compared to the HPV positive with normal cytology group. Reassurance was similarly high across these three normal results groups. Tables 4 and 5 provide an overview of the results for all secondary outcome measures.

## Discussion

Informing women that they test positive for high‐risk HPV accompanied by any cytology result appears to be associated with some adverse psychological effects at the population level, at least in the short‐term. Our findings are consistent with previous studies showing that testing positive for HPV with abnormal cytology is associated with raised anxiety and distress.^8^, ^9^, ^10^, ^11^ However, unique to HPV primary screening and unique to the literature, we also found evidence of raised anxiety, concern about the screening result and worry about developing cervical cancer in women who tested positive for HPV with normal cytology. These women were more anxious than the control group (normal cytology; no HPV test) and displayed a mean anxiety score slightly above the upper threshold expected in the general population (mean score of 38.3 compared to the normal range of 34–36).^23^, ^24^ They were also 1.9 times more likely to exhibit very high anxiety compared to the control group (indicated by a STAI score > 49/80), scoring similarly to individuals with clinically important symptoms or an anxiety disorder.^25^, ^26^, ^27^ Women who test positive for HPV with normal cytology carry a very low absolute risk of developing high‐grade cervical abnormalities or cancer in the near future.^28^, ^29^ Therefore, for many women, informing them of this test result may lead to unnecessary adverse psychological responses. At the population level, it is unlikely that the levels of anxiety observed in our study would cause significant disruptions to women's daily functioning. This is supported by our small between‐group differences for general distress paired with wider evidence indicating that screening‐related anxiety is usually temporary.^12^, ^13^, ^30^ However, it is important to remember that 72% of women aged 25–64 living in the UK attend for screening when invited,^1^ of whom 8.5% are likely to be HPV positive with normal cytology.^7^ Given the very large numbers of women affected, it is imperative that we do not lose sight of subgroups of individuals who may be more at risk of acute adverse reaction (e.g., very high anxiety). Clinically significant levels of anxiety may be more common in women who do not understand their result^10^, ^12^, ^15^; however, research is needed to establish the risk factors and trajectory of high anxiety following an HPV positive result to inform efforts to mitigate this adverse response.

Reassuringly, women with persistent HPV and normal cytology at 12 months did not have significantly higher anxiety than the control group, although descriptively they displayed slightly higher anxiety than would be expected in the general population (mean score of 36.8 compared to normal range of 34–36). This suggests that raised levels of anxiety and distress associated with an initial HPV positive result may normalise with repeated exposure to the result and/or over time, which is consistent with previous research.^13^, ^30^ Our findings suggest that efforts to reduce anxiety should therefore primarily focus on women who test HPV positive with normal cytology for the first time.

HPV primary screening has a high negative predictive value and therefore has the potential to reassure the majority of women who are at extremely low immediate risk of cervical cancer. Our findings indicate that testing HPV negative at any point (including 12 months after an HPV positive result) is associated with high levels of reassurance. However, although an HPV negative result offers better protection from cervical cancer than normal cytology,^6^, ^31^, ^32^ women in our study felt similarly reassured after receiving an HPV negative result compared to normal cytology. Low knowledge of HPV and the benefits associated with an HPV negative result may partially account for this.^33^ Normal or “good” results may also demand little cognitive attention and therefore reduce the likelihood of differentiation.^34^, ^35^

### Strengths and limitations

To the best of our knowledge, this is the first major study to evaluate the short‐term psychological impact of primary HPV testing within a routine national programme. Participant recruitment linked to routine clinical management through the NHSCSP HPV primary screening pilot ensured accurate data collection and broad geographical coverage across England. A control group with primary cytology allowed additional between‐group comparisons, strengthening our cross‐sectional design. Our sample size was smaller than we anticipated for the group who cleared HPV at 12‐months (*n* = 66); however, this group had similar scores to the other normal and HPV negative groups. Our response rate was 21% which raises uncertainty regarding the extent to which our sample is representative of the wider screening population. We were, however, able to statistically weight our data to the wider screening population for age and IMD quintile as well as compare demographic characteristics between responders and nonresponders. For consistency with the previous NHSCSP evaluation of HPV triage methods,^10^, ^13^ some of our secondary outcomes were single‐item, nonvalidated measures. Finally, like many cross‐sectional survey studies, we had an underrepresentation of nonwhite participants, and self‐selection bias may have resulted in an overrepresentation of the most anxious women.

### Implications

Cervical screening programmes should aim to mitigate unnecessary anxiety, worry and concern in women testing positive for HPV with any cytology result. Use of clear, evidence‐based communication in test result letters and information materials will help ensure that women understand their results and the implications for cancer risk. Provision of communication skills training for sample takers related to areas which are anticipated to increase women's anxiety (e.g., fear of cancer, sexual implications, transmission) should help minimise adverse psychological responses. Reasons for the switch to HPV primary screening should also be clearly communicated to the public ahead of implementation, to reduce the risk of a public backlash like the one recently observed in Australia,^36^ where a minority of individuals believed that switching to HPV primary screening would miss some cervical cancers. So far, there does not seem to have been any opposition to HPV primary screening in the English pilot sites, suggesting that current communication efforts are working effectively. The findings of our study should help to inform national screening implementation policies and evaluations in other countries where HPV primary screening is being implemented. Future research should explore what makes women most anxious and determine the strongest modifiable predictors of anxiety (and very high anxiety) in women testing HPV positive, to inform the development of interventions.

## Conclusions

Testing positive for HPV with normal or abnormal cytology was associated with short‐term adverse psychological effects in routine HPV primary screening, although it is unlikely that this will lead to significant disruption of daily functioning for most women. Our cross‐sectional comparison of women receiving their first *vs*. second HPV positive with normal cytology test result suggests that anxiety is likely to be short‐lived and does not persist for women on 12‐month early recall.

## Author contributions

Study concept: Waller J, Marlow L, Forster A, Kitchener H, Patnick J. Measurement development: Waller J, Marlow L, McBride E, Forster A. Project management: McBride E, Waller J. Experimental analyses: Ridout D, McBride E. Drafted the manuscript: McBride E, Waller J. Final version of the manuscript: all authors. The corresponding author attests that all listed authors meet authorship criteria and that no others meeting the criteria have been omitted.

## Supporting information


**Table S1** Demographic characteristics of nonresponders *vs*. responders (no weights or adjustments applied)
**Table S2.** Results of univariate analysis for primary and secondary outcomes by test result groups (unweighted and using raw data only)

## Data Availability

The datasets generated and/or analysed during the study are available from the corresponding author on reasonable request.
