# Supplementary material for: Anxiety and distress following receipt of results from routine HPV primary testing in cervical screening: The psychological impact of primary screening (PIPS) study
Source: Int J Cancer. 2019 Jul 23;146(8):2113–21. doi: 10.1002/ijc.32540 (PMC7065242; doi:10.1002/ijc.32540)
Supplement: Supplementary file 1 — Table S1 Demographic characteristics of nonresponders vs. responders (no weights or adjustments applied) Table S2. Results of univariate analysis for primary and secondary outcomes by test result groups (unweighted and using raw data only) [file IJC-146-2113-s001.docx]

**Supplementary Table 1. Demographic characteristics of non-responders vs. responders (no weights or adjustments applied)**

|  | **Non-responders** | **Responders** |  |
| --- | --- | --- | --- |
| **N** | 4346 | 1148 |  |
| **Age**  N  Mean years (SD) | 4344  38.3 (10.9) | 1124  41.2 (11.8) | t(5466)=-7.7 *p*=<.001 |
| **IMD Quintile (n, %)**  1 (most deprived)  2  3  4  5 (least deprived) | 1079 (25.6%)  926 (22.0%)  935 (22.2%)  692 (16.4%)  575 (13.7%) | 170 (16.3%)  211 (20.2%)  276 (26.5%)  193 (18.5%)  193 (18.5%) | *X*²(4)=53.9, *p*=<.001 |
| **No. of previous screens**  N  Mean screens (SD) | 4248  5.0 (4.6) | 1077  6.3 (4.9) | t(5323)=-8.3 *p*=<.001 |
| **NHS site (n, %)**  Liverpool  Sheffield  London North West  Norfolk & Norwich  Manchester | 669 (15.4%)  794 (18.3%)  789 (18.2%)  425 (9.8%)  1169 (38.4%) | 185 (16.1%)  216 (18.8%)  150 (13.1%)  205 (17.9%)  392 (34.1%) | *X*²(4)=70.3, *p*=<.001 |
| **Test Result (n, %)**  Control (no HPV test)  HPV negative  HPV positive, normal cytology  HPV positive, abnormal cytology  HPV persistent  HPV cleared | 1121 (25.8%)  980 (22.5%)  935 (21.5%)  639 (14.7%)  479 (11.0%)  192 (4.4%) | 211 (18.4%)  249 (21.7%)  263 (22.9%)  171 (14.9%)  184 (16.0%)  70 (6.1%) | *X*²(5)=46.0, *p*=<.001 |

The end column displays the results of Chi-squared analysis and t-tests comparing non-responders to responders.

**Supplementary Table 2 – Results of univariate analysis for primary and secondary outcomes by test result groups (unweighted and using raw data only)**

|  | **Control**  **(no HPV test)** | **HPV negative** | **HPV positive, normal cytology** | **HPV positive, abnormal cytology** | **HPV persistent at 12-months** | **HPV cleared at 12-months** |
| --- | --- | --- | --- | --- | --- | --- |
| **Anxiety**  MD (95% CI)  p-value | ref | -2.0 (-4.5, 0.61)  0.14 | 3.4 (0.8, 6.0)  0.01 | 7.2 (4.4, 10.1)  <.001 | 1.9 (-0.9, 4.7)  0.19 | 2.1 (-1.8, 6.0)  0.29 |
| **Distress**  MD (95% CI)  p-value | ref | -0.4 (-1.0, 0.3)  0.26 | 0.4 (-0.2, 1.1)  0.16 | 1.0 (0.3, 1.7)  <.01 | - 1. (-0.5, 0.9)   0.60 | 0.2 (-0.7, 1.2)  0.66 |
| **Very high anxiety**  Odds Ratio (95% CI)  p-value | ref | 1.0 (0.6, 1.7)  0.96 | 1.8 (1.1, 3.1)  0.02 | 3.5 (2.0, 5.9)  <.001 | 1.4 (0.8, 2.5)  0.27 | 1.4 (0.7, 3.1)  0.36 |
| **Case-level distress**  Odds Ratio (95% CI)  p-value | ref | 0.9 (0.6, 1.4)  0.55 | 1.2 (0.8, 1.8)  0.40 | 1.5 (0.9, 2.3)  0.11 | 1.2 (0.8, 2.0)  0.35 | 1.0 (0.5, 1.9)  0.97 |
| **Worry about cancer**  Odds Ratio (95% CI)  p-value | ref | - 1. (0.5, 1.5)   0.69 | 4.6 (2.9, 7.3)  <.001 | 5.0 (3.0, 8.1)  <.001 | 4.6 (2.8, 7.4)  <.001 | 1.2 (0.5, 2.5)  0.70 |
| **High concern**  Odds Ratio (95% CI)  p-value | 0.07 (0.03, 0.2)  <.001 | 0.06 (0.03, 0.1)  <.001 | ref* | 1.8 (1.2, 2.7)  < 0.01 | 0.9 (0.6, 1.4)  0.79 | 0.1 (0.03, 0.3)  <.001 |
| **High reassurance**  Odds Ratio (95% CI)  p-value | 13.5 (7.9, 23.0)  <.001 | 12.1 (7.5, 19.7)  <.001 | ref* | 1.1 (0.8,1.7)  0.55 | 1.1 (0.8, 1.7)  0.51 | 6.2 (3.2, 12.2)  <.001 |

MD = mean difference; 95% CI = 95% confidence intervals; p = <.05 interpreted as statistically significant.

Ref = reference group. *The reference group for concern and reassurance is HPV positive with normal cytology due to very low and very high proportions (respectively) of positive responses in the control group for these two outcomes.
